# A two-rescuer-method significantly alters CPR-quality during cardiopulmonary resuscitation in an airliner cabin - a randomized, controlled manikin trial

**DOI:** 10.1038/s41598-025-30996-1

**Published:** 2025-12-10

**Authors:** Jan Schmitz, Christoph Ernst, Lydia Johnson Kolaparambil Varghese, Steffen Kerkhoff, Gerrit Jansen, Jochen Hinkelbein

**Affiliations:** 1 Department of Anesthesiology and Intensive Care Medicine, Medical Faculty, University Hospital of Cologne, 50937 Cologne, Germany; 2 German Society of Aerospace Medicine, Munich, Germany; 3 Department of Anesthesiology and Intensive Care Medicine, Klinikum Leverkusen, Leverkusen, Germany; 4 Department of Anaesthesiology, Intensive Care Medicine and Emergency Medicine, Johannes Wesling Klinikum Minden, University Hospital, Ruhr- University Bochum, Minden, Germany; 5 Department of Anesthesiology, Intensive Care Medicine and Emergency Medicine, Marienhaus Klinikum St. Elisabeth Neuwied, 56564 Neuwied, Germany

**Keywords:** CPR, Manikin, In-Flight medical emergency, Resuscitation, Preclinical research, Myocardial infarction, Ventricular fibrillation, Heart failure, Trauma, Hypoxia

## Abstract

Between 1/15,000–1/50,000 passengers suffer in-flight medical emergencies (IFME) with cardiac arrest accounting for 0.3 %. Confined space can have a negative impact on quality of chest compressions during cardiopulmonary resuscitation (CPR), thus we have conducted a randomized controlled study to find the most effective approach of performing CPR in a one – vs. two-rescuer method in a simulated airliner cabin. We randomized 20 healthcare professionals to perform a set of 10 min Basic Life Support (BLS, chest compressions and bag-mask-ventilation) in a one- vs. two-rescuer scenario and in confined space vs. open space in a randomized order using a full-body manikin. The primary outcome was compression depth as sensitive marker for differences in CPR-quality. The study was registered on clinicaltrials.gov (NCT02002481). Mixed ANOVAs with post-hoc false-discovery-rate adjusted pairwise comparisons indicated that one- vs. two-rescuer method showed differences in no-flow-time (confined: 8.05 ± 0.17 vs. 24.25 ± 1.05 s/2min and open space: 7.51 ± 0.02 vs. 21.31 ± 0.43 s/2min; p < 0.001) and missing releases (confined: 27.09 ± 5.55 vs. 46.64 ± 9.66 number/10 minutes and open space: 27.09 ± 2.44 vs. 43.36 ± 6.4 number/10minutes; p < 0.001). A confined space significantly elevated no-flow-time in the two-rescuer-method vs. the one-rescuer-method (24.24 ± 1.06 s/2min vs. 21.26 ± 0.44 s/2min; p < 0.001), whereas compression frequency and compression depth were different but still within the current recommendations of ERC/AHA in both methods per condition. Limited space in an airliner cabin has significant impact on no-flow-time in a two-rescuer-method. In case of CPR and limited access to the patient, we recommend a one-rescuer-method as first approach to ensure early and high-quality CPR for experienced personnel.

## Introduction

Approximately 4.5 billion passengers worldwide travel by commercial airlines per year and the trend is significantly increasing^1^. Although cardiac arrest during air travel is relatively rare (0.3% of emergencies, approximately 800 incidents per year), the unusual setting, limited resources, and especially the confined space to perform cardiopulmonary resuscitation (Basic and Advanced Life Support, BLS/ALS) represent an unique challenge for healthcare providers^2^.

Standard cardiopulmonary resuscitation is recommended to be performed in a two-rescuer-method, one kneeling next to the patient suffering from cardiac arrest applying chest compression, the other one kneeling in a head position to perform bag-mask-ventilation in a 30:2 ratio and switching positions every two minutes^3^. In certain circumstances, it may be difficult or impossible to perform CPR in this position, for example in confined spaces such as a narrow corridor in an aircraft cabin. In the confines of an aircraft aisle, it may be difficult to perform standard CPR with one or two rescuers kneeling at the side of the patient, especially when switching positions every two minutes as it is recommended in current guidelines^3–5^.

An alternative for a single-rescuer setting is to compress and ventilate whilst kneeling in a head position as it is also recommended in different guidelines^2,5,6^. Over-the-head-CPR (OTH-CPR) as a one-rescuer-method appears as effective as a two-rescuer-CPR^7–9^. OTH cardiopulmonary resuscitation is a method of chest compression, which may be easier to perform than standard CPR in a confined space. Different authors showed, that there are no differences between the kinematics, compression forces, depths, and frequencies obtained using a single-rescuer-method as practiced by experienced providers^10,11^. For less experienced providers current guidelines emphasize the importance of switching compression and ventilation positions every two minutes, as previous data have shown that physical exhaustion after two minutes determines high-quality (compression frequency and depth) CPR^10,11^.

In the confined and resource-limited environment of an aircraft cabin, however, providing effective ventilation (by mouth-to-mouth/nose or bag-mask) presents considerable difficulties^5,12^. These practical barriers have contributed to a global shift in terrestrial BLS guidelines toward prioritizing chest compressions over ventilations, particularly in adult cardiac arrest^3^.

While basic life support can usually be delivered effectively in an aircraft cabin using available equipment and trained cabin crew, advanced life support (ALS) presents a far greater set of challenges. These differences arise from the complexity of ALS procedures, the limitations of the onboard environment, and the restricted medical resources available during flight^5^.

Airline cabin crew in the European Union (EU) are trained in Basic Life Support in accordance with the guidelines of the European Resuscitation Council (ERC), performing CPR in a two-rescuer-method with added oxygen for ventilation^6^.

The aim of this study was to assess possible differences in CPR-quality between a simulated airliner cabin and without limited space and to give specific recommendations to ensure high-quality CPR in case of in-flight cardiac arrest (IFCA).

## Materials and methods

### Ethics

The study protocol was approved by the ethics committee of the University Hospital of Cologne (Cologne, Germany, number 11–289, 08/12/2015) and conducted according to the principles of the Declaration of Helsinki and Good Clinical Practice. Written informed consent was obtained from all participants before enrollment. The study was registered on clinicaltrials.org (NCT02002481, 05/12/2013).

### Participants

Twenty male and female participants were invited for medical eligibility screening. To minimize pre-existing differences in CPR quality due to experience and training, we only included participants with board certified medical education as physician and relevant experience in treating cardiac arrest (> 3 years of active occupation in emergency medical services).

After completing a short questionnaire to gather information about general demographics and the physical condition, participants underwent medical examination. To be eligible, participants needed to be board-certified physicians between the age of 18 and 60, free from any acute or chronic health issues that could affect CPR performance, particularly cardiopulmonary conditions. The exclusion criteria included any acute or chronic disease, any internal or surgical medical condition with impact on CPR performance, permanent medication use (except birth-control-pills), acute infectious disease, any psychiatric conditions, or pregnancy. Available literature indicates that oral contraceptive use has negligible impact on parameters such as inflammatory markers, cardiovascular function, cognitive performance, or general metabolic indices in healthy participants.

Participants were recruited using stratified random sampling to achieve demographic balance with respect to age and sex. The target population was divided into four strata according to sex (male, female) and age group (< 30 years, ≥ 30 years). Within each stratum, participants were randomly selected until the desired total sample size of 20 was reached. This method was employed to reduce sampling bias and improve representativeness given the limited sample size.

Participants were selected and randomized into two arms by 1:1 ratio using an online randomizer software (Research Randomizer, https://www.randomizer.org/).

### Protocol

**Fig. 1:**
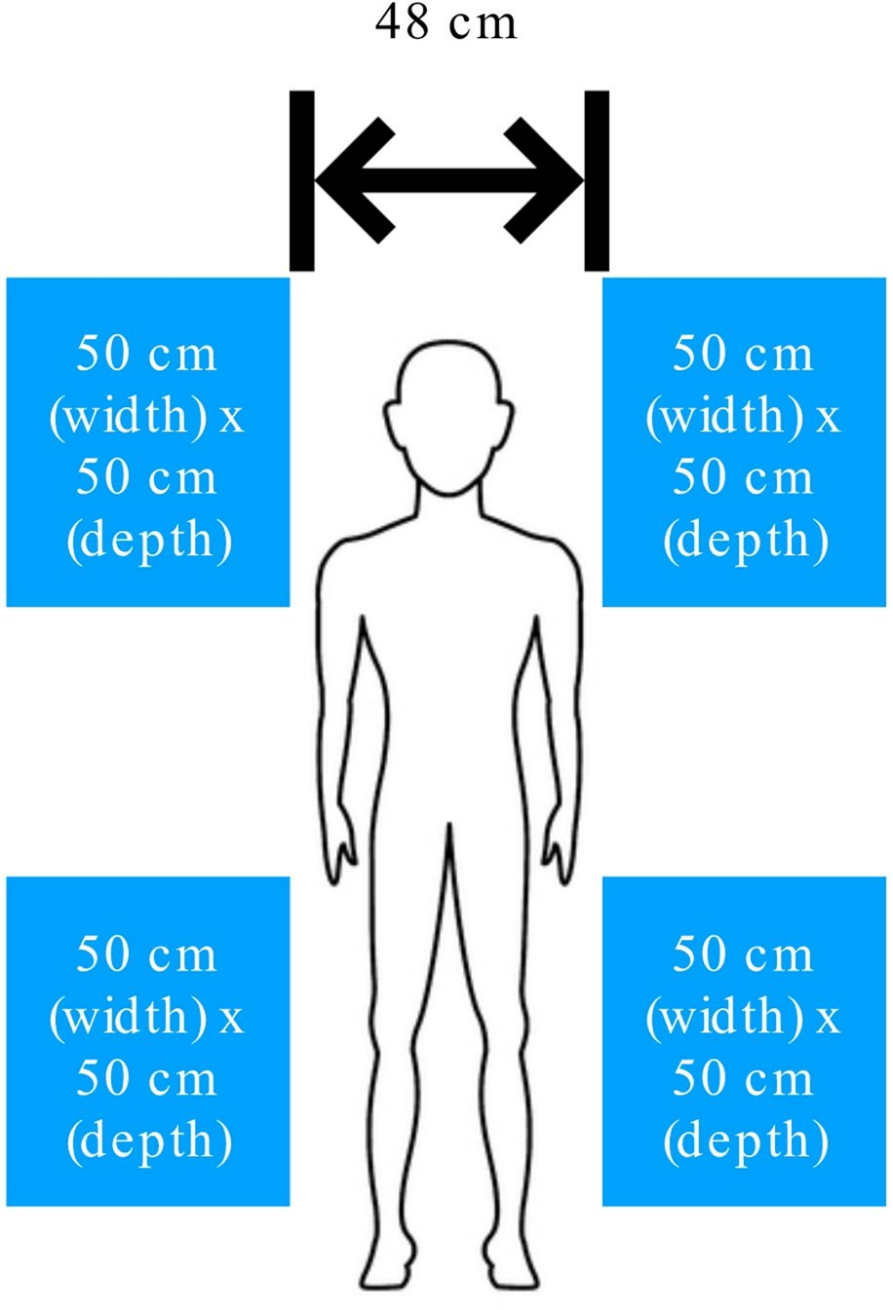
Schematic illustration of the study setting.

We conducted the study in the premises of a flight simulator (www.youfly.de), simulating an airliner cabin with original seats in a realistic scenario (seat width: 50 cm, aisle width: 48 cm), located near Cologne, Germany. A schematic illustration of the study setting is shown in Fig. 1. The results are reported in accordance with the CONSORT guidelines^13^ and the protocol is included as supplemental material.

After a physical examination and giving written informed consent, participants were randomized into two pathways, performing a set of 10 min Basic Life Support (BLS) in a confined vs. open space and in a one-vs.- a two-rescuer method, consisting of 30 chest compressions, followed by two bag-mask-ventilations. Airline protocols vary widely and focus primarily on recognizing cardiac arrest and providing chest compressions, but also emphasize the need for ventilation. Flight attendants are generally not trained in the use of bag-mask ventilation, but it is legally required that a bag-mask be carried on board the aircraft. To ensure a realistic basic life support scenario, our protocol followed a 30 compressions to 2 ventilations ratio. Participants in the two-rescuer-scenario had to coordinate themselves within the scenario.

**Fig. 2:**
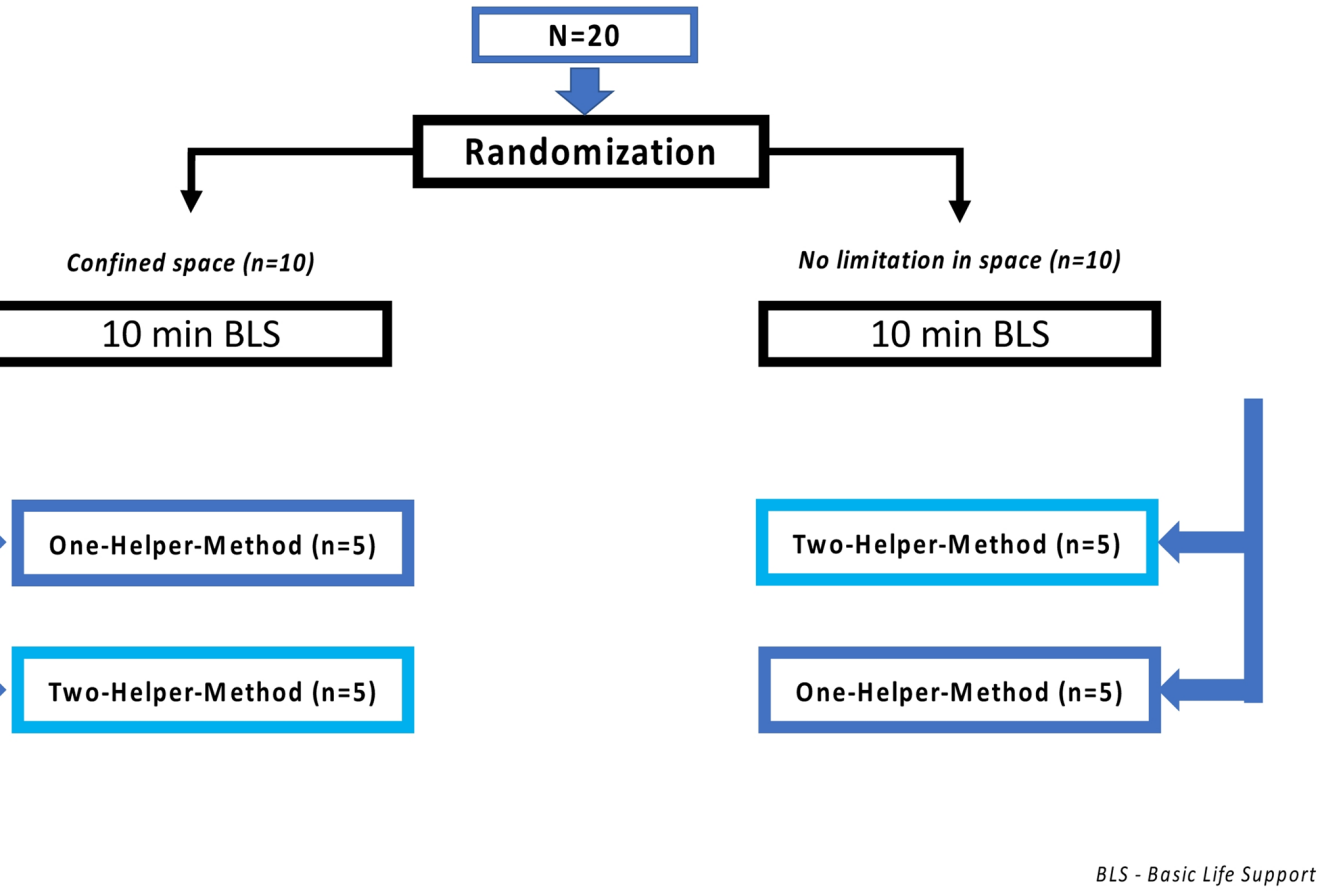
Flowchart of study design.

During each set, a minimum 30 min recreational phase was provided for physical recovery. Other environmental factors (i.e., noise, lighting) were maintained the same in all conditions. External study personnel operated the full-body manikin. A detailed description of the study design is shown in Fig. 2.

### Data collection

Chest compressions and ventilations were performed using a full-body manikin (AmbuMan^®^ Airway Wireless, Ambu Ltd., Bad Nauheim, Germany) on the ground with sufficient light. Participants performed Basic Life Support in a confined space (explained above) and in open space of minimum 4 m^2^. The AmbuMan^®^ Airway Wireless application (Ambu^®^, Ambu Ltd., Bad Nauheim, Germany) was run on a Laptop (Microsoft^®^ Cooperation, Redmond, Washington, United States of America) monitoring data-driven information and accurate measurements of relevant CPR parameters as well as storage of individual CPR data.

### Data availability

All data generated or analyzed during this study are included in this published article.

### Statistics

A statistical power analysis (GPower 3.1 software) was performed a priori for sample size estimation. Assuming a mean difference of 3% in compression depth with a standard deviation of 3.3% (Cohens d = 0.9) [at t = minute 8–10], *n* = 10 individuals needed to be analyzed to achieve 80% power based on a paired t-test (two-sided) and alpha-level of 5%.

Data was processed with Excel for Mac 16.32 (Microsoft^©^, Redmond, USA) and statistical analyses were performed with RStudio^©^ software (version 2022.07.1).

The demographic parameters sex, weight, height, and age were compared between groups using independent t-tests.

The primary endpoint was:

- mean compression depth (mm) at condition confined vs. open space.

The secondary endpoints were:

- ventilation (number of applied ventilations/min).
- breathing volume (litre/breath).
- no-flow-time (seconds/2 min block).
- compression frequency (number/minute).
- missing releases (number/10 minutes).

We used random subject mixed ANOVAs with the factors “condition” (confined space, open space), “timepoint” (minute 0–2, minute 2–4, minute 4–6, minute 6–8, minute 8–10) and the interaction between “condition x timepoint” for analyses of endpoints. The factor ‘timepoint’ was secondly used as parameter for possible exhaustion over the period of resuscitation. Post-hoc pairwise comparisons (in total 24 planned comparisons of interest: two within conditions (2 × 3 = 6), and five between conditions at each timepoint (3 × 5 = 15)) were adjusted for multiple comparisons with the False-Discovery-Rate. Transformations were used to achieve normal distribution of residuals.

Results were considered significant if *p* < 0.05. All findings are presented as means ± standard deviation (p-value) if not stated otherwise.

## Results

### Participants

Demographic parameters (11 male and 9 female, age 35.5 ± 8.6 years, BMI 23.6 ± 2.7 kg/m^2^) did not differ between the groups.

### Influence of confined vs. open space on CPR-quality

Confined space significantly elevated no-flow-time in the two-rescuer-method (24.24 ± 1.06 s/2min vs. 21.26 ± 0.44 s/2min; *p* < 0.001). The One-rescuer-Method showed no difference in CPR quality in a confined vs. open space. Every parameter representing CPR-quality was within the recommendation of ERC/AHA. Table 1 shows CPR parameters for CPR-quality in a confined space vs. open space for a comparison of a single-rescuer-approach per condition and a two-rescuer-method per condition.

### Influence of one- vs. two-rescuer method on CPR-quality in a confined and vs. open space

Comparison of one- vs. two-rescuer method showed significant influence on no-flow-time (confined: 8.05 ± 0.17 vs. 24.25 ± 1.05 s/2min and open space: 7.51 ± 0.02 vs. 21.31 ± 0.43 s/2min; *p* < 0.001) and missing releases (confined: 27.09 ± 5.55 vs. 46.64 ± 9.66 number/10 minutes and open space: 27.09 ± 2.44 vs. 43.36 ± 6.4 number/10minutes; *p* < 0.001) in both the conditions.

Also, compression depth was significantly lower in the two-rescuer-method but still within the current recommendations of ERC/AHA. Table 2 shows CPR parameters for CPR-quality of a single-rescuer-method per condition vs. a two-rescuer-method per condition.

**Table 1:** Confined vs. open space in space CPR-parameters as one-rescuer-method (above) and two-rescuer-method (bottom).

Parameter	Space											ANOVA (*p*-values)		
	Confined						Open Space							
***One-rescuer-method***	*Minute 0–2*	*Minute 2–4*	*Minute 4–6*	*Minute 6–8*	*Minute 8–10*	*Minute 0–2*		*Minute 2–4*	*Minute 4–6*	*Minute 6–8*	*Minute 8–10*	*Timepoint*	*Condition*	*Interaction*
Ventilation (number/min)	4.43 ± 1.16	4,63 ± 1.16	4.65 ± 0.1	4,83 ± 0.8	4,63 ± 0.8	4,6 ± 1.31		4.93 ± 1.31	4.7 ± 1.3	5.1 ± 1.02	5 ± 0.95	0.940	0.052	0.817
Breathing volume (l/breath)	0.38 ± 0.05	0.4 ± 0.05	0.4 ± 0.05	0.4 ± 0.05	0.4 ± 0.05	0.41 ± 0.05		0.4 ± 0.11	0.4 ± 0.1	0.4 ± 0.06	0.41 ± 0.05	0.482	0.387	0.481
No-Flow-Time (sec/2 min)	7.82 ± 1.08	8.23 ± 1.31	8.17 ± 1.3	8.07 ± 1.14	7.95 ± 1.24	7.52 ± 1		7.48 ± 0.69	7.52 ± 0.82	7.5 ± 0.8	7.51 ± 0.9	0.921	0.074	0.354
Compression rate (number/min)	121.15 ± 11.52	118.3 ± 13.21	118.05 ± 13.11	117.25 ± 14.07	118 ± 13.7	123 ± 12.32		121 ± 13.01	121.8 ± 16.09	120.4 ± 12.64	120.1 ± 12.62	0.631	0.510	0.590
Compression depth (mm/compression)	61.85 ± 8.02	60.16 ± 8.45	60.79 ± 9.05	60.7 ± 9.11	60.18 ± 9.59	62.09 ± 7.01		61.31 ± 7.51	60.53 ± 7.66	60.05 ± 8.26	59.87 ± 8.25	0.441	0.988	0.468
Missing releases (total number/10min)	36.35 ± 53.62	23.65 ± 47.77	24.5 ± 47.97	22.85 ± 45.72	28.1 ± 51.71	30.25 ± 50.51		25.85 ± 47.66	24.85 ± 47.45	25.35 ± 47.34	29.15 ± 49.49	0.052	1.000	0.517

**Parameter**	**Space**											**ANOVA (p-values)**		
	**Confined**						**Open Space**							
***Two-rescuer-method***	*Minute 0–2*	*Minute 2–4*	*Minute 4–6*	*Minute 6–8*	*Minute 8–10*	*Minute 0–2*		*Minute 2–4*	*Minute 4–6*	*Minute 6–8*	*Minute 8–10*	Timepoint	Condition	Interaction
Ventilation (number/min)	5.45 ± 1.12	6.1 ± 1.61	6 ± 0.78	6.3 ± 0.86	5.85 ± 1.11	5.85 ± 1		6.85 ± 0.58	6.6 ± 0.88	5.85 ± 0.71	5.65 ± 1.47	**0.048**	0.206	0.588
Breathing volume (l/breath)	0.41 ± 0.06	0.4 ± 0.62	0.41 ± 0.54	0.4 ± 0.54	0.39 ± 0.57	0.389 ± 0.53		0.42 ± 0.58	0.41 ± 0.39	0.4 ± 0.56	0.42 ± 0.62	0.652	0.874	0.076
No-Flow-Time (sec/2 min)	22.6 ± 2.26	25.18 ± 2.18	24 ± 1.93	25.13 ± 2.46	24.33 ± 2.8	21.1 ± 1.64		21.04 ± 2.04	21.26 ± 1.72	22.03 ± 2.51	20.93 ± 2	0.091	**< 0.001**	0.347
Compression rate (number/min)	120.7 ± 6.72	121.9 ± 7.59	121.2 ± 10.17	119.5 ± 8.07	121.2 ± 8.79	122.2 ± 9.33		121.1 ± 8.12	120.9 ± 6.06	120.8 ± 6.96	120.7 ± 10.29	0.585	0.946	0.585
Compression depth (mm/compression)	59.84 ± 6.27	59.92 ± 4.12	58.14 ± 4.56	59.93 ± 5.02	58.63 ± 2.2	59.39 ± 5.57		58.57 ± 5	59.75 ± 3.99	58.8 ± 4.22	61.04 ± 4.5	0.826	0.905	0.068
Missing releases (total number/10min)	44.9 ± 57.33	41.3 ± 51.88	34.3 ± 51.91	56.9 ± 59.28	55.8 ± 67.36	53.5 ± 53.67		43.1 ± 53.93	36 ± 39.78	40.8 ± 54.93	43.4 ± 55.92	0.281	0.886	0.471

**Table 2:** One-vs.- two-rescuer method in a confined (top) vs. open space (bottom).

Parameter in a confined space	Method	Minute of BLS					ANOVA (*p*-values)		
		*Minute 0–2*	*Minute 2–4*	*Minute 4–6*	*Minute 6–8*	*Minute 8–10*	*Timepoint*	Condition	*Interaction*
Ventilation (number/min)	One-rescuer-method	4.43	4.63	4.65	4.83	4.63	**< 0.001**	**< 0.001**	**< 0.001**
	Two-rescuer-method	5.45	6.10	6.00	6.30	5.85			
Breathing volume (l/breath)	One-rescuer-method	0.38	0.40	0.40	0.40	0.40	0.2123	0.1261	0.1789
	Two-rescuer-method	0.41	0.40	0.41	0.40	0.39			
No-Flow-Time (sec/min)	One-rescuer-method	7.82	8.23	8.17	8.07	7.95	**< 0.001**	**< 0.001**	**< 0.001**
	Two-rescuer-method	22.60	25.18	24	25.13	24.33			
Compression rate (number/min)	One-rescuer-method	121.15	118.30	118.05	117.25	118	0.0431	0.0336	0.0213
	Two-rescuer-method	120.70	121.90	121.90	119.50	121.20			
Compression depth (mm/compression)	One-rescuer-method	61,85	60.16	60.79	60.70	60.18	0.0234	0.0307	0.0301
	Two-rescuer-method	59.84	59.92	58.14	59.93	58.83			
Missing releases (total number/10min)	One-rescuer-method	36.35	23.65	24.50	22.85	28.10	**< 0.001**	**< 0.001**	**< 0.001**
	Two-rescuer-method	44.90	41.30	34.30	56.90	55.80			

**Parameter in open space**	**Method**	**Minute of BLS**					**ANOVA (p-values)**		
		*Minute 0–2*	*Minute 2–4*	*Minute 4–6*	*Minute 6–8*	*Minute 8–10*	Timepoint	Condition	Interaction
Ventilation (number/min)	One-rescuer-method	4.60	4.93	4.70	5.10	5.00	0.0088	0.0336	0.1112
	Two-rescuer-method	5.85	6.85	6.60	5.85	5.65			
Breathing volume (l/breath)	One-rescuer-method	0.41	0.4	0.4	0.4	0.41	0.4138	0.0459	0.0918
	Two-rescuer-method	0.38	0.42	0.41	0.4	0.42			
No-Flow-Time (sec/min)	One-rescuer-method	7.52	7.48	7.52	7.5	7.51	**< 0.001**	**< 0.001**	**< 0.001**
	Two-rescuer-method	21.3	21.04	21.26	22.03	20.93			
Compression rate (number/min)	One-rescuer-method	123	121	121.8	120.4	120.1	0.3827	0.4186	0.4750
	Two-rescuer-method	122.2	121.2	120.9	120.8	120.7			
Compression depth (mm/compression)	One-rescuer-method	62.09	61.31	60.53	60.05	59.87	0.0778	0.1729	0.3680
	Two-rescuer-method	59.39	58.57	59.75	58.8	61.04			
Missing releases (total number/10min)	One-rescuer-method	30.25	25.85	24.85	25.35	29.15	< 0.001	< 0.001	< 0.001
	Two-rescuer-method	53.5	43.1	36	40.8	43.4			

## Discussion

This study represents the first attempt to provide specific recommendations for in-flight CPR under conditions of limited patient access. Our findings indicate that limited space in an airliner cabin has primarily significant impact on no-flow-time in a two-rescuer-method, thus we recommend using a one-rescuer method in scenarios where access to the patient is constrained to ensure high-quality CPR.

Clinical investigation during study conduct showed, that the two rescuers had trouble to switch positions every two minutes as recommended by current ERC/AHA guidelines for resuscitation^3,4^. Isle width and seat-to-seat distance were major limitations to ensure fast and secure switch of position. To our knowledge, no previous study has evaluated the problem of switching positions in a two-rescuer-method within a confined space so far, although it has already been recommended to consider a one-rescuer-method in case of limited patient access^10,14,15^.

Interestingly, our first endpoint (compression depth) was within BLS-guidelines throughout the study, which underlines no exhaustion during the time period of consecutive ten minutes chest compression, but no-flow-time was a more sensitive parameter in this study, as it was also already shown in other studies comparing one-vs. two-rescuer-scenarios^16^.

Our second comparison focused on the differences between a one-rescuer-vs. a two-rescuer-method in both the conditions of (non-) limited patient access. Previous studies have shown, that a one-rescuer-method appears as effective as a two-rescuer-method with some marginal advantages in correct hand placement, as different studies have been demonstrated previously^7–9^.

Our results showed, that a confined space increased no-flow-time significantly, especially when using the two-rescuer-method. Also, a slight effect on other CPR-quality parameters can be discussed, as compression frequency and compression depth were influenced negatively in the two-rescuer-method in a confined space. A possible explanation could be a higher level of stress because of trouble during position switch every two minutes as other studies may have also suggested before^16,17^.

The fact of organizing the position switch with increased demand of communication may shift focus away from maintaining compression frequency and -depth. Also previous studies showed negative influences of stress on CPR-quality, as external distractors markedly reduced the quality of cardiopulmonary resuscitation^18^. It can be postulated, that CPR during air travel has a great potential to distract from focusing on maintaining CPR performance by well trained personnel in terms of increased noise, bad lighting and other environmental factors^5,19,20^.

## Limitations

An important limitation of our study is that CPR was conducted under laboratory conditions with a manikin. This setup may not produce the psychological stress and neurohumoral activation of real CPRs on a commercial airline flight^20–22^. Moreover, we did not simulate the limited ambient oxygen, noise and vibrations inside an aircraft cabin, which might also influence CPR performance. Furthermore, we conducted our study with healthcare professionals experienced in CPR, which cannot be easily translated to less experienced (or lay-) persons conducting CPR during an in-flight emergency. Nonetheless, existing data shows that the probability of a healthcare professional presence onboard is quite high and due to a standardized BLS protocol our findings may also be transferred to other healthcare professionals (e.g., nurses or paramedics). During the ten minutes set of BLS it is probably easier for experienced health care providers to adapt to the situation of confined environmental space and to reduce possible effects of limited space. Another limitation in this context is, that this study only gathers data of the first ten minutes of BLS, as realistic scenarios of in-flight cardiac arrest probably require a longer period of attempting resuscitation efforts.

Our results should be verified in further studies in a more realistic simulated cabin environment. Our findings are limited to the five timepoints that we scheduled for CPR. It remains unclear, if beginning alterations of compression frequency and compression depth are more expressed in more realistic conditions of an airliner cabin (e.g. with supplemental hypoxia during cruise). We suggest to examine these questions in specifically designed future studies.

## Conclusion

This study was the first to show, that a confined space simulating an airliner cabin has significantly elevated no-flow-time in the two-rescuer-method vs. the one-rescuer-method, whilst compression frequency and compression depth were different but still within the current recommendations of ERC/AHA.

In case of In-Flight-CPR and limited access to the patient, we recommend a one-rescuer-method as first approach to ensure early and high-quality CPR for trained personnel. After acclimatization to the BLS-scenario conducting a one-rescuer-method and organizing further medical and personal resources, ground-based BLS/ALS approaches should be implemented as soon as possible.

## Data Availability

All data generated or analyzed during this study are included in this published article.
